# Nationwide Implementation of HIV Molecular Cluster Detection by Centers for Disease Control and Prevention and State and Local Health Departments, United States

**DOI:** 10.3201/eid3113.241143

**Published:** 2025-05

**Authors:** Anne Marie France, Camden J. Hallmark, Nivedha Panneer, Rachael Billock, Olivia O. Russell, Mary Plaster, Jessica Alberti, Fathima Nuthan, Neeraja Saduvala, David Philpott, M. Cheryl Bañez Ocfemia, Scott Cope, Angela L. Hernandez, Sergei L. Kosakovsky Pond, Joel O. Wertheim, Steven Weaver, Saja Khader, Kevin Johnson, Alexandra M. Oster

**Affiliations:** US Public Health Service Commissioned Corps, Atlanta, Georgia, USA (A.M. France, A.M. Oster); Centers for Disease Control and Prevention, Atlanta (A.M. France, C.J. Hallmark, N. Panneer, R. Billock, O.O. Russell, D. Philpott, M.C.B. Ocfemia, S. Cope, A.L. Hernandez, A.M. Oster); DLH Corporation, Atlanta (M. Plaster, J. Alberti, F. Nuthan, S. Khader); SeKON Enterprise Inc., Atlanta (N. Saduvala); Temple University, Philadelphia, Pennsylvania, USA (S.L. Kosakovsky Pond, S. Weaver); University of California San Diego, La Jolla, California, USA (J.O. Wertheim, S. Weaver); Oak Ridge Institute for Science and Education, Oak Ridge, Tennessee, USA (K. Johnson)

**Keywords:** HIV, molecular cluster, HIV transmission, molecular epidemiology, outbreak detection, HIV/AIDS and other retroviruses, sexually transmitted infections, viruses, United States

## Abstract

Detecting and responding to clusters of rapid HIV transmission is a core HIV prevention strategy in the United States, guiding public health interventions and identifying gaps in prevention and care services. In 2016, the Centers for Disease Control and Prevention (CDC) initiated molecular cluster detection using data from 27 jurisdictions. During 2016–2023, CDC expanded sequence reporting nationwide and deployed Secure HIV-TRACE, an application supporting health department (HD) molecular cluster detection. CDC conducts molecular cluster detection quarterly; state and local HDs analyze local data monthly. HDs began routinely reporting clusters to CDC by using cluster report forms in 2020. During 2018–2023, CDC identified 404 molecular clusters of rapid HIV transmission; 325 (80%) involved multiple jurisdictions. During 2020–2023, HDs reported 298 molecular clusters to CDC; 249 were first detected by HDs. Expanding molecular cluster detection has provided a foundation for improving service delivery to networks experiencing rapid HIV transmission.

HIV clusters or outbreaks are defined as rapid HIV transmission among persons in a sex or drug-using network (*1*); network refers to persons in an HIV cluster and those with whom they have sex or share drugs, who might or might not have HIV. Identifying rapid HIV transmission through cluster detection guides public health efforts designed to identify and address gaps in care and prevention services that are not effectively reaching HIV transmission networks (*2*). Before 2016, HIV clusters in the United States were detected sporadically, typically by astute medical providers or partner services and frontline staff (*2*).

In 2016, after responding to a large outbreak of HIV among persons who inject drugs in Scott County, Indiana, USA (*3*), the Centers for Disease Control and Prevention (CDC) initiated proactive cluster detection through routine analysis of CDC’s National HIV Surveillance System (NHSS) data. HIV is a nationally notifiable disease condition, and state, tribal, local, and territorial (STLT) health departments (HDs) collect demographic, transmission risk, and clinical information and report deidentified data to CDC. NHSS data are routinely used at federal and STLT levels to monitor HIV distribution and transmission, plan and evaluate prevention and care programs, allocate resources, inform policy development, and identify and respond to rapid transmission across the United States (*4*). Laboratory reporting, including HIV molecular viral sequence data generated through routine clinical HIV drug-resistance testing, is an integrated component of NHSS.

Surveillance-based cluster detection methods include approaches to identify geospatial increases in HIV diagnoses (*5*), referred to as time-space analysis, as well as molecular cluster detection, in which clusters are recognized through analysis of HIV sequence data. HIV mutates rapidly, and molecular cluster detection methods assess differences between virus nucleotide sequences (genetic distance) to distinguish infections that are more closely related in transmission networks from those more distantly related. Time-space analysis and molecular cluster detection, in addition to cluster detection by providers or partner services and frontline staff, are complementary (*1*).

Many approaches are available for HIV sequence analysis, but not all approaches focus on clusters with the highest transmission rates, which contribute disproportionately to current and future transmission (*6*,*7*). CDC developed an approach focusing on clusters representing rapid transmission (*8*), which yields clusters having transmission rates >8 times the overall national transmission estimate among all persons living with HIV (*8*) and some clusters with rates >33 times the national rate (*9*).

Molecular cluster detection at both local and national levels is critical. Local HIV surveillance data are available during collection, but analyses by HDs are limited to persons diagnosed or living within the HD’s administrative boundary who have had data reported to the local surveillance system. However, populations are mobile (*10*), and HIV transmission can be geographically dispersed (*11*). National HIV surveillance data are not as timely but can be analyzed by CDC to identify clusters that cross jurisdictional boundaries (*8*,*12*).

CDC initiated routine analysis of HIV surveillance data in 2016 to identify clusters at the national level, including clusters spanning multiple jurisdictions. Those analyses included sequence data reported to CDC by 27 STLT HDs to assess drug resistance and general transmission patterns (*13*). The HIV TRAnsmission Cluster Engine (HIV-TRACE) (*14*), a tool developed to assess global HIV transmission patterns (*15*), was adapted to characterize transmission patterns in a local installation within CDC that adhered to stringent HIV data protections (*13*). However, secure local installation and implementation of HIV-TRACE was technically complex and not feasible for most STLT HDs. In 2018, cluster detection and response (CDR) was expanded nationwide (*16*).

To promote analysis of molecular HIV data to elucidate transmission patterns at STLT levels, CDC initiated the development of Secure HIV-TRACE in 2015 through funding from CDC’s Advanced Molecular Detection program, part of the National Center for Emerging and Zoonotic Infectious Diseases. It was essential to ensure that this web-based tool met stringent HIV data protections. This secure, web-based bioinformatics application incorporates sequence analysis methods similar to HIV-TRACE. Secure HIV-TRACE was first released for HD use in 2017 for characterizing general transmission patterns. After CDR expansion nationwide in 2018 (*16*), Secure HIV-TRACE was refined to focus on detecting and monitoring clusters of rapid HIV transmission. Secure HIV-TRACE is computationally efficient, scales to accommodate large datasets and was designed for use by public health staff who might lack bioinformatics expertise.

With expanded cluster detection capabilities both at national and STLT levels and the recognition of the importance of cluster response to reduce HIV incidence, CDR was included as 1 of 4 pillars of the federal Ending the HIV Epidemic Initiative launched in 2019 (*17*). We describe the implementation of molecular HIV cluster detection at both the national and STLT levels and assess the contributions of each to overall cluster detection in the United States.

## Materials and Methods

### Data Collection

HDs report HIV surveillance data to CDC according to STLT laws and regulations. HIV surveillance programs collect demographic, clinical, laboratory, vital statistics, and behavioral data. NHSS data collection includes HIV laboratory results indicative of HIV infection, such as CD4+ T lymphocyte numbers, viral load test results, and HIV sequence data. HIV sequences are collected from genotypic resistance tests performed as part of routine clinical care at commercial, private, and public health laboratories and reported electronically to STLT HDs (*4*).

During 1997–2012, sequences were collected through supplemental surveillance projects focused on drug resistance and virus diversity, expanding from 4 jurisdictions in 1997 to 17 in 2012 (*18*). During 2013–2017, reporting of HIV sequence data expanded to 27 jurisdictions with the additional aim to assess transmission patterns and, in 2018, expanded to 59 HD HIV surveillance programs with the charge to use those data for HIV CDR (*16*).

### National Cluster Detection

A CDC-provided software application (Enhanced HIV/AIDS Reporting System [eHARS]) is installed at HDs for entry, storage, management, and reporting of HIV data conducted by surveillance programs; the application is secured behind each HD’s firewall and accessible by designated HD staff only. HDs transfer monthly deidentified HIV surveillance data to CDC. Each quarter, CDC produces national-level datasets that deduplicate and reconcile data for persons reported by all jurisdictions into a single, unified dataset for reporting, analyses, and evaluation (*4*). Data are protected by a CDC Assurance of Confidentiality (*1*), and release is governed according to the data rerelease agreement with each HD. For annual HIV surveillance reports, data are considered preliminary until a 12-month reporting delay has elapsed. After 12 months, data are considered provisional and are subject to change as additional data are reported (*4*).

National cluster detection is conducted after each quarterly preliminary national-level dataset is created by using all available HIV sequences of suitable quality, which can include multiple sequences per person. Cluster detection is conducted using preliminary datasets to expedite timely detection of clusters. At each quarterly interval, clusters of rapid transmission among persons with HIV diagnosed in the previous 3 years are identified by using a secure local installation of HIV-TRACE (*14*); this transmission network analysis includes a 1,497-nt segment of the HIV protease and reverse transcriptase genomic region. Sequences are aligned with the HIV-1 *pol* gene in reference strain HXB2, pairwise genetic distances are calculated, and clusters are defined when the pairwise genetic distance is <0.005 substitutions/site (i.e., meeting a 0.5% genetic distance threshold) (*8*). HIV clusters meeting CDC’s national priority definition are then identified.

CDC defines national priority clusters to focus on clusters having evidence of rapid transmission by using the 0.5% genetic distance threshold and data for persons with an HIV diagnosis within the previous 3 years. Such clusters with >3 HIV diagnoses in the previous 12 months have high transmission rates (*8*). If that definition was used nationwide, many jurisdictions would have more priority clusters than they could feasibly conduct response activities for; thus, that definition is applied to low-burden jurisdictions (those with <200 diagnoses annually), whereas a higher threshold of >5 diagnoses is used for most jurisdictions nationally. All clusters meeting those definitions are considered national priority clusters.

Each quarter, new national priority clusters are identified and growth in previously identified clusters is monitored. Clusters that continue to meet or newly meet national priority criteria are flagged for review. Because of disruptions related to the COVID-19 pandemic, national cluster detection was not conducted for the March 2020 quarter.

Many national priority clusters cross jurisdictional boundaries. The primary jurisdiction for each cluster is defined as the jurisdiction where >50% of cluster members resided at the time of HIV diagnosis. In 2022, CDC began systematically identifying jurisdictions with substantial involvement in national priority clusters; substantial involvement is defined as >3 diagnoses (resident at diagnosis or current resident) in the previous 12 months. Multiple jurisdictions can be substantially involved in a cluster at the same time.

Each quarter, summary reports of new and previously identified national priority clusters are generated for jurisdictions. Those reports include current case counts, jurisdictions involved, and current priority status. In addition, line lists are generated for newly detected clusters and clusters that continue to meet priority criteria and have grown. Those data are securely transmitted to HDs. HDs are asked to review the information in conjunction with results of their local molecular cluster analysis. CDC epidemiologists meet with HD personnel, as needed, to discuss findings and offer technical assistance to respond to clusters.

### State and Local Cluster Detection

Beginning in 2018, CDC expanded support to all HDs to collect HIV sequence data and conduct molecular cluster detection monthly (*16*). Methods for state and local molecular cluster detection parallel those used for national cluster detection; however, several critical differences exist (Table 1). To provide an accessible tool for HDs to conduct molecular analysis, CDC contracted with the University of California San Diego (La Jolla, CA, USA) and Temple University (Philadelphia, PA, USA) to develop Secure HIV-TRACE. Although the original intent was to characterize transmission patterns, the development and intended use evolved to focus on detecting and monitoring HIV molecular clusters. Funding for Secure HIV-TRACE development was obtained through CDC’s Advanced Molecular Detection program (fiscal years 2015–2017) and the Division of HIV Prevention, National Center for HIV, Viral Hepatitis, STD, and TB Prevention, since that time.

**Table 1:** Comparison of HIV cluster detection by CDC and state and local health departments, United States*

Comparisons	CDC	State/local
Interval	Quarterly	At least monthly
Data	National, deduplicated	State or local
Ability to detect multijurisdictional clusters	Yes	Not at the time of writing
Analytic tool	Local installation of HIV-TRACE	Secure HIV-TRACE
Identifiable information	No personal identifiers	Linked to personal identifiers†
Initial notification to health departments	CDC securely transmits notification of priority clusters to state/local health departments	Secure HIV-TRACE automatically flags priority clusters identified in each analysis
Reporting from health departments to CDC	Response activities reported to CDC via cluster report forms	Response activities reported to CDC via cluster report forms

*CDC, Centers for Disease Control and Prevention; HIV-TRACE, HIV TRAnsmission Cluster Engine. †Identifiers are not uploaded to Secure HIV-TRACE or transmitted to CDC.

To conduct molecular cluster detection, HDs export data from their local eHARS, process the data by using a CDC-supplied SAS software program (SAS Institute Inc., https://www.sas.com), and securely upload data without personal identifiers to Secure HIV-TRACE. Similar to the national analysis, Secure HIV-TRACE aligns the sequences to the HIV-1 *pol* gene from reference strain HXB2, computes pairwise genetic distances, and defines clusters at the 0.5% and 1.5% genetic distance thresholds. Secure HIV-TRACE users can visualize data and review summary statistics, epidemiologic curves, and line-listed information that can be exported for further analysis.

**Figure 1:**
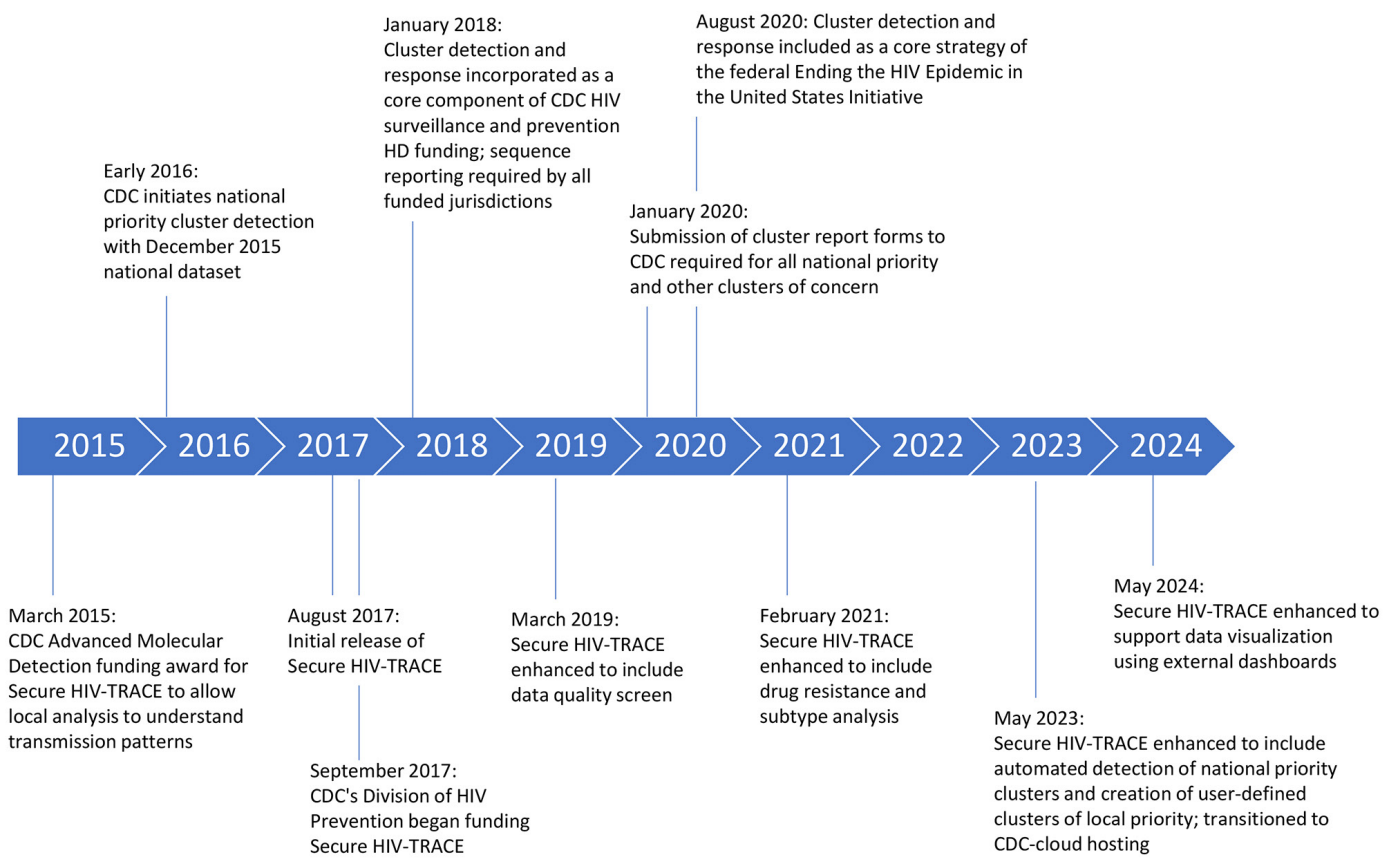
Timeline of nationwide implementation of HIV cluster detection and response by CDC and state and local HDs in the United States. CDC, Centers for Disease Control and Prevention; HD, health department; Secure HIV-TRACE, Secure HIV TRAnsmission Cluster Engine.

When first released, Secure HIV-TRACE focused broadly on transmission networks, using a 1.5% genetic distance threshold to define clusters. Ten additional releases since 2017 have expanded application functionality; cluster detection has been aligned with CDC’s focus on clusters of rapid transmission by using the national priority cluster definition with a 0.5% genetic distance threshold. In May 2023, Secure HIV-TRACE was enhanced to automatically identify and monitor growth in clusters meeting CDC’s national priority criteria. Before that enhancement, a CDC-supplied SAS program was used to identify national priority clusters according to Secure HIV-TRACE output. Users can now also elect to monitor other clusters of interest on the basis of locally defined priority criteria. Additional enhancements have included a data quality screen, HIV drug resistance and subtype analysis, improved visualization, and optional data visualizations using external platforms such as Power BI (Microsoft, https://www.microsoft.com) (Figure 1). Secure HIV-TRACE hosting was also modernized by a move to the CDC cloud platform with access through CDC’s Secure Access Management Services.

### Cluster Report Forms

Since 2020, CDC-funded STLT HDs have been required to submit HIV cluster report forms each quarter to CDC to report clusters of public health concern, including molecular clusters meeting CDC’s national priority cluster criteria and other clusters of concern detected through both molecular and other approaches. Cluster report forms promote communication between HDs and CDC and have information about methods of cluster detection, key cluster attributes, and cluster response activities (*19*). Using a secure REDCap (https://www.project-redcap.org) platform, HDs submit initial cluster report forms, which have information on how the cluster was first detected, cluster size at detection, and other characteristics. Follow-up forms are submitted during the response to report ongoing response activities and findings, and annual/closeout forms are submitted at the end of the response (or annually for ongoing responses) to provide additional activity and outcome information.

When cluster report forms are submitted, jurisdictions are also asked to enter cluster-related variables in eHARS for persons with HIV who are known to be part of the cluster. Cluster report forms and cluster-related variables do not include personal identifiers. The variables include cluster identification numbers assigned locally and national cluster identification numbers for those clusters also identified through national cluster detection. That information enables HDs and CDC to elucidate characteristics and care status of persons with HIV who are part of reported clusters and help guide response activities.

### Analysis

We assessed the number of diagnoses for which HIV sequence data were reported and described characteristics of clusters detected through national molecular analysis during 2018–2023. We defined sequence completeness as the percentage of persons with a reported HIV diagnosis for which a sequence of >100 bp was reported. To characterize clusters identified through state/local cluster detection, we analyzed data reported on cluster report forms from 2020–2023.

## Results

### Sequence Reporting

Using data reported to CDC’s NHSS until December 2023, we determined sequences were available for 52% of HIV diagnoses during 2021–2023, an increase from 41% sequence completeness observed for the 3-year period of 2016–2018 (as reported by December 2018). During 2023, the median timeframe from collection of the sample to receipt of the sequence at the HD was 34 (interquartile range 20–50) days.

### National Molecular Analysis

During 2018–2023, CDC detected 404 national priority clusters (Table 2). An average of 67 (range 48–77) new clusters were detected each year; the fewest were detected during the COVID-19 pandemic peak in 2020, when only 3 quarterly analyses were conducted. The median number of persons in each cluster at the time of detection was 7 (range 3–24). Clusters had grown to a median size of 13 (range 3–193) by December 2023; clusters detected in earlier years (and therefore with more time for potential growth) had higher median sizes as of December 2023. Of the 404 clusters, 74 (18%) continued to meet national priority criteria in December 2023, indicating that the cluster had >5 diagnoses during 2023 or >3 diagnoses in low-burden jurisdictions. Of 59 CDC-funded HDs, 43 (73%) were the primary jurisdiction for >1 cluster.

**Table 2:** Number of HIV molecular clusters detected each year through nationwide analysis of National HIV Surveillance System data by Centers for Disease Control and Prevention, United States, 2018-2023*

Characteristics	2018	2019	2020†	2021	2022	2023	Total no.
National priority clusters detected‡	69	74	48	61	77	75	404
Clusters meeting national priority criteria as of December 2023‡	2 (3)	8 (11)	5 (10)	9 (15)	10 (13)	40 (53)	74 (18)
Median cluster size when first detected (range)	7 (3–24)	8 (3–19)	8 (3–16)	7 (4–18)	7 (3–14)	7 (3–23)	7 (3–24)§
Median cluster size as of December 2023 (range)	17 (3–193)	17.5 (3–90)	20 (3–49)	16 (6–72)	11 (3–47)	9 (3–26)	13 (3–193)§
Clusters involving >1 jurisdiction as of December 2023	58 (84)	62 (84)	43 (90)	51 (84)	56 (73)	55 (73)	325 (80)
Clusters with no primary jurisdiction at detection	6 (9)	9 (12)	4 (8)	5 (8)	5 (6)	6 (8)	35 (9)
Jurisdictions with >1 cluster as the primary jurisdiction at detection¶	25	25	25	20	26	28	43#
Jurisdictions with >1 cluster with substantial involvement**	NA	NA	NA	NA	27	31	NA
Clusters with no jurisdiction ever substantially involved	NA	NA	NA	NA	2 (3)	5 (7)	NA
Clusters with only 1 jurisdiction ever substantially involved	NA	NA	NA	NA	68 (88)	67(89)	NA
Clusters with >2 jurisdictions ever substantially involved	NA	NA	NA	NA	7 (9)	3 (4)	NA

*Values are no. (%) except as indicated. NA, not applicable. †National cluster detection was not conducted for the March 2020 quarter because of disruptions related to the COVID-19 pandemic. ‡National priority clusters are defined as clusters at the 0.5% genetic distance threshold with >5 diagnoses in the previous 12 months or >3 diagnoses in the previous 12 months in low-burden jurisdictions (those having <200 reported HIV diagnoses annually). National priority criteria is assessed on an ongoing basis. §Overall median and range. ¶Primary jurisdiction is defined as the jurisdiction in which >50% of cluster members resided at the time of HIV diagnosis. If there is no single jurisdiction in which >50% of cluster members reside at diagnosis, no primary jurisdiction is assigned. #Total number of unique jurisdictions. **Substantial involvement in national priority clusters is defined as >3 diagnoses in a given jurisdiction (resident at diagnosis or current resident) in the previous 12 months. Multiple jurisdictions can be substantially involved in a cluster at the same time.

Most clusters spanned multiple jurisdictions; 325 (80%) clusters involved >1 jurisdiction, including clusters with >1 diagnosis outside the primary jurisdiction and those with no primary jurisdiction. Among 152 clusters identified since substantial involvement was first systematically assessed beginning in 2022, 7 (5%) had no jurisdiction, 135 (89%) had only 1 jurisdiction, and 10 (7%) had >2 jurisdictions substantially involved.

### State and Local Cluster Detection

Secure HIV-TRACE was first released in August 2017 as an optional tool for jurisdictions collecting sequence data at that time. In January 2018, the application became available to all jurisdictions as HIV CDR expanded. Use of Secure HIV-TRACE expanded over time. In January 2018, 30 HDs had been using Secure HIV-TRACE. By April 2024, 148 HD users representing 54 HDs had been using Secure HIV-TRACE and had uploaded 792,360 sequences to the application for cluster detection analysis.

**Figure 2:**
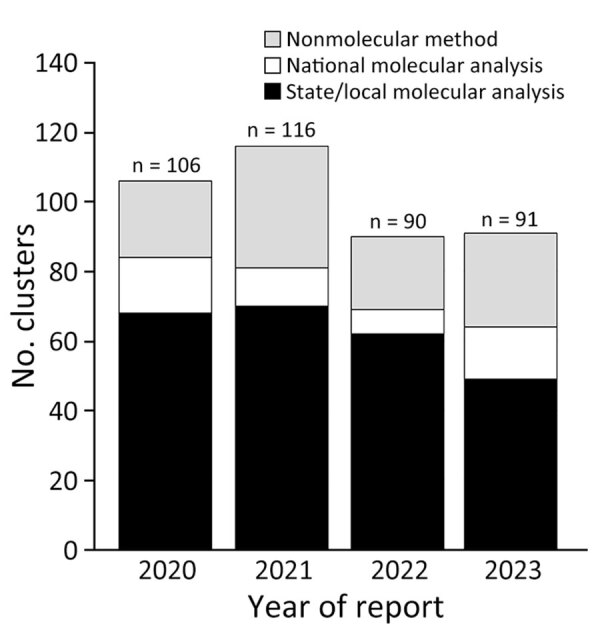
Clusters of HIV newly reported to the Centers for Disease Control and Prevention by state and local health departments, United States, during 2020–2023. Clusters were reported to Centers for Disease Control and Prevention through cluster report forms. Methods by which clusters were first detected are indicated; nonmolecular cluster detection methods include time-space cluster detection, partner services, and provider notification. Numbers on top of bars indicate exact number of HIV clusters reported each year.

**Figure 3:**
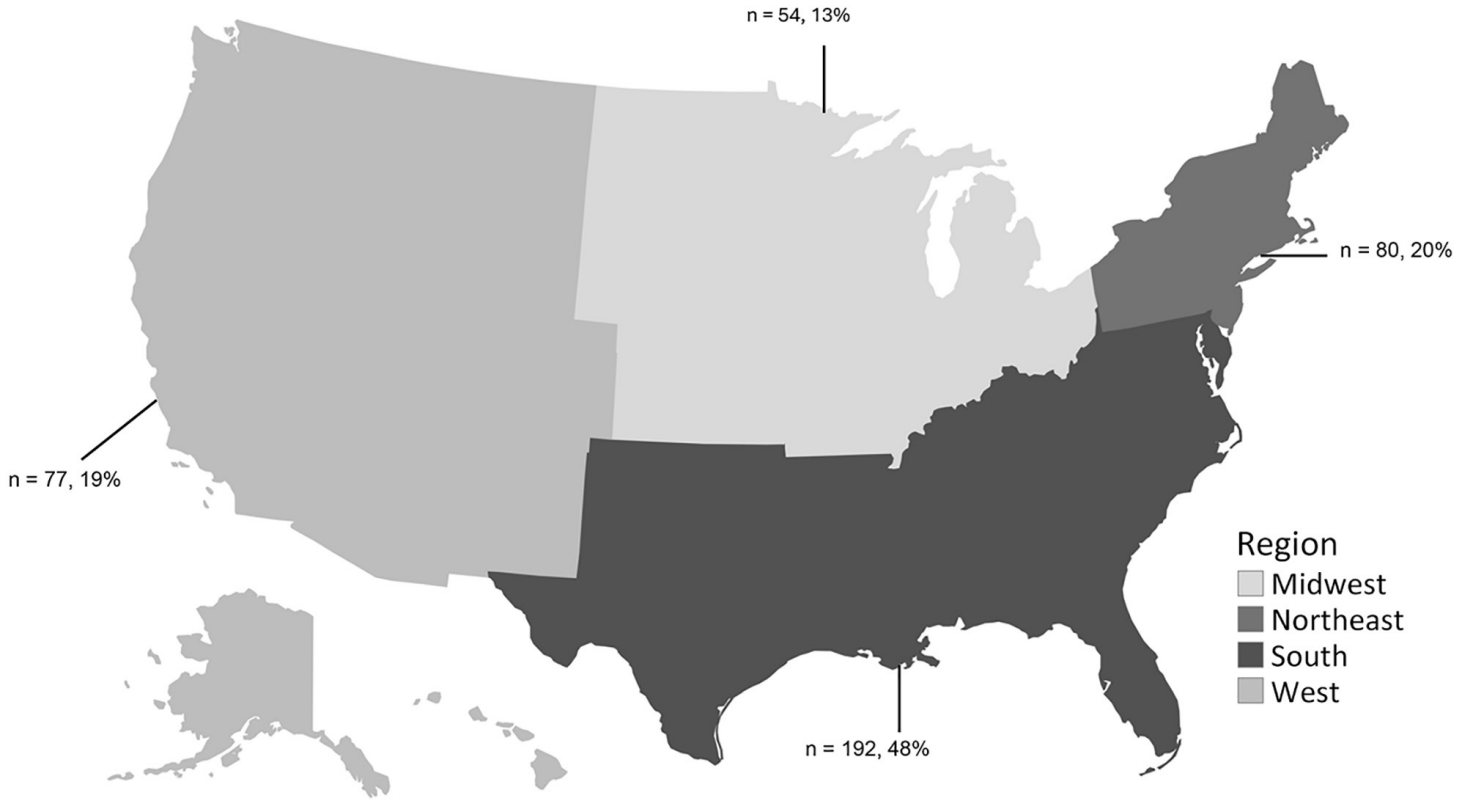
Region of reporting health department for clusters of HIV newly reported to the Centers for Disease Control and Prevention by state and local health departments, United States, during 2020–2023. Clusters were reported to Centers for Disease Control and Prevention through cluster report forms. Numbers and percentages of clusters are indicated for each region.

During 2020–2023, a total of 403 (90–116/year) clusters newly detected through any method were reported by HDs to CDC via cluster report forms (Figure 2). Of those, 298 (74%) were first detected through national or state/local molecular analysis; 248 (83%) of 298 were first detected through state/local analysis. Clusters were most often (48%) reported by jurisdictions in the South of the United States (Figure 3). The median size of molecular clusters first detected through state/local analysis was 7 (range 2–85) at detection, and 224 (90%) of 248 met national priority criteria when first reported. Of 59 CDC-funded jurisdictions, 37 (62%) reported >1 cluster first detected through state/local molecular analysis.

## Discussion

CDC and state and local HDs have successfully implemented routine detection and monitoring of clusters of rapid HIV transmission through analysis of molecular sequence data. Clusters of rapid HIV transmission are now frequently detected in the United States; both national and state/local molecular cluster analyses are essential for HIV cluster detection. Most jurisdictions have had >1 national priority cluster identified through CDC analysis, and most jurisdictions have reported a molecular cluster to CDC that was first identified through state/local molecular analysis. The higher frequency of reported clusters in the South is consistent with the greater burden of new diagnoses in that region (*20*). Multijurisdictional clusters are commonly identified, and clusters often exhibit ongoing growth many years after detection.

National and state/local cluster detection are complementary, both contributing to comprehensive and timely cluster identification. Because NHSS datasets are only available quarterly, monthly state/local cluster detection is essential for more timely cluster detection. Most molecular clusters reported to CDC by HDs have first been detected through state/local analysis, promoting a timely response. In addition, state and local analyses enable flexibility to detect clusters not yet meeting priority criteria but still of concern because of local epidemiology or other factors. Secure HIV-TRACE is the primary tool for state and local cluster detection. Although the tool used for cluster detection is not explicitly reported by HDs on cluster report forms, nearly all reported clusters detected through state/local molecular analysis have likely been detected by using Secure HIV-TRACE. That secure, accessible tool has been essential for implementing cluster detection, and tool enhancements have improved identification and monitoring of clusters meeting national or local priority.

State and local HIV surveillance systems in the United States are decentralized, and state/local cluster detection analyses by HDs are limited to data from their own jurisdictions. Therefore, national-level cluster detection conducted by CDC is essential for detection of multijurisdictional clusters; 20% of nationally identified clusters are missed by local cluster detection (*21*). HIV is a chronic infection, and persons living with HIV in the United States are more mobile than the general US population (*10*). The frequent identification of multijurisdictional clusters is consistent with findings that transmission networks are often geographically dispersed (*11*). Multijurisdictional involvement can range from a single diagnosis in an otherwise geographically focused cluster to clusters with no geographic focus. The substantial involvement definition captures evidence of rapid transmission occurring in multiple jurisdictions, suggesting the need for meaningful response engagement and coordination from the jurisdictions involved.

Traditional approaches to identifying rapid transmission, including time-space analysis, rely on observing increases in diagnoses in a population or area. However, those approaches are limited by factors such as a median HIV diagnosis delay of 3 years in the United States (*22*) and difficulty detecting increases in diagnoses in areas with higher baseline HIV incidence. In addition, populations are mobile (*10*), and detecting geographically dispersed rapid transmission is difficult using traditional approaches (*11*). Molecular cluster detection can detect rapid transmission that is geographically dispersed or in areas with a high baseline HIV incidence. Robust HIV surveillance systems are critical for effective cluster detection.

For cluster detection to have the intended effect, response is essential. Rapid transmission occurs because affected communities are not adequately reached by existing services (*1*). Clusters affect various populations and can grow rapidly (*23*,*24*); most clusters are associated with sexual transmission (*24*). Responses to clusters can vary depending on local priorities, resources, and needs identified through cluster investigation and might include individual, network, and structure-level interventions to ensure that testing, care, and prevention programs are reaching persons and places that can most benefit. Response activities and outcomes have been previously described (*2*,*19*).

Evidence that HIV CDR strengthens HIV prevention and care services has been observed in a growing body of field investigations, as well as analytic and modeling studies (*2*,*6*,*7*,*25*). Priority clusters correspond to underlying networks that are 3–9 times the size of the detected cluster; those networks have high transmission rates and disproportionate numbers of persons with undiagnosed HIV infections, indicating opportunities for public health intervention (*25*). Clusters can grow rapidly (*23*) and contribute disproportionately to future infections (*6*,*7*).

Community engagement is essential to implement HIV CDR. Although analysis of molecular data provides a unique and powerful tool to identify communities affected by rapid HIV transmission, some advocates and community members have raised concerns about collection and analysis of molecular sequence data, including concerns about the potential use of molecular HIV data in criminal transmission cases (*26*,*27*). CDC has held numerous discussions with community members, community-based organizations, advocates, and other key partners to inform responsible establishment of CDR activities and guide the use of sequence data (*27*,*28*). CDC has strong security measures to ensure privacy and confidentiality of persons with HIV, requires health departments to comply with data security and confidentiality guidelines, and has further strengthened guidance for protection of sequence data (*29*,*30*). Secure HIV-TRACE is not an open database. The deidentified individual-level information submitted to NHSS is not publicly available. Given the potential harms of disclosure, CDC’s data use agreement prohibits release without individual-level consent. CDC also requires HDs to communicate and collaborate with community members and partners for input on CDR activities and to design responses to specific clusters and outbreaks, including, where needed, enhancing or improving processes or procedures for protecting privacy and confidentiality (*1*,*30*,*31*). That engagement helps HDs address community concerns, provides a foundation for trust and collaboration, and supports collaboration with community partners for cluster response.

We are in an era where we have the tools to successfully treat and prevent HIV. However, those tools often do not reach the persons who need them most. Molecular cluster detection enables us to identify rapid HIV transmission that would have previously been unrecognized. That detection creates new opportunities to identify and close gaps in HIV prevention and care services and advances efforts to end the HIV epidemic in the United States.
